# Disaggregating Data to Measure Racial Disparities in COVID-19 Outcomes and Guide Community Response — Hawaii, March 1, 2020–February 28, 2021

**DOI:** 10.15585/mmwr.mm7037a1

**Published:** 2021-09-17

**Authors:** Joshua J. Quint, Miriam E. Van Dyke, Hailey Maeda, J. Keʻalohilani Worthington, May Rose Dela Cruz, Joseph Keaweʻaimoku Kaholokula, Chantelle Eseta Matagi, Catherine M. Pirkle, Emily K. Roberson, Tetine Sentell, Lisa Watkins-Victorino, Courtni A. Andrews, Katherine E. Center, Renee M. Calanan, Kristie E.N. Clarke, Delight E. Satter, Ana Penman-Aguilar, Erin M. Parker, Sarah Kemble

**Affiliations:** ^1^Hawaii State Department of Health; ^2^Epidemic Intelligence Service, CDC; ^3^CDC COVID-19 Response Team; ^4^Office of Public Health Studies, University of Hawaiʻi at Mānoa, Honolulu, Hawaii; ^5^Department of Native Hawaiian Health, University of Hawaiʻi at Mānoa, Honolulu, Hawaii; ^6^Office of Hawaiian Affairs; ^7^Office of Minority Health and Health Equity, CDC; ^8^Office of Tribal Affairs and Strategic Alliances, CDC.

Native Hawaiian and Pacific Islander populations have been disproportionately affected by COVID-19 (*1**–**3*). Native Hawaiian, Pacific Islander, and Asian populations vary in language; cultural practices; and social, economic, and environmental experiences,^†^ which can affect health outcomes (*4*).^§^ However, data from these populations are often aggregated in analyses. Although data aggregation is often used as an approach to increase sample size and statistical power when analyzing data from smaller population groups, it can limit the understanding of disparities among diverse Native Hawaiian, Pacific Islander, and Asian subpopulations^¶^ (*4*–*7*). To assess disparities in COVID-19 outcomes among Native Hawaiian, Pacific Islander, and Asian populations, a disaggregated, descriptive analysis, informed by recommendations from these communities,** was performed using race data from 21,005 COVID-19 cases and 449 COVID-19–associated deaths reported to the Hawaii State Department of Health (HDOH) during March 1, 2020–February 28, 2021.^††^ In Hawaii, COVID-19 incidence and mortality rates per 100,000 population were 1,477 and 32, respectively during this period. In analyses with race categories that were not mutually exclusive, including persons of one race alone or in combination with one or more races, Pacific Islander persons, who account for 5% of Hawaii’s population, represented 22% of COVID-19 cases and deaths (COVID-19 incidence of 7,070 and mortality rate of 150). Native Hawaiian persons experienced an incidence of 1,181 and a mortality rate of 15. Among subcategories of Asian populations, the highest incidences were experienced by Filipino persons (1,247) and Vietnamese persons (1,200). Disaggregating Native Hawaiian, Pacific Islander, and Asian race data can aid in identifying racial disparities among specific subpopulations and highlights the importance of partnering with communities to develop culturally responsive outreach teams^§§^ and tailored public health interventions and vaccination campaigns to more effectively address health disparities.

Descriptive data of Hawaii state residents reported to HDOH during March 1, 2020–February 28, 2021, were analyzed to determine the number, percentage, and crude rates of COVID-19 cases and deaths using race categories that were not mutually exclusive. Data were analyzed among the five minimum racial origin categories defined by the Office of Management and Budget (American Indian or Alaska Native, Asian, Black or African American, Native Hawaiian or Other Pacific Islander, and White), and among Native Hawaiian, Pacific Islander, and Asian origin subcategories.^¶¶^ Ethnicity was not included in this analysis because data on ethnicity were missing for 32% of reported cases and 9% of deaths. Race information for COVID-19 patients was mostly self-reported; race information for deaths was reported by patients premortem or by an observer (e.g., physician) or a proxy family member. Because a large proportion of Hawaii’s population identifies as multiracial,*** analyses were conducted with groups that were not mutually exclusive, including persons of one race alone or in combination with one or more races (*6*). Using this approach, persons of more than one race were counted multiple times, depending upon the number of race groups recorded. Thus, race categories (e.g., Native Hawaiian and Pacific Islander and Asian) and subcategories (e.g., Marshallese and Filipino) include persons with any mention of those races.

Among 25,480 COVID-19 cases and 450 COVID-19–associated deaths reported in Hawaii during March 2020–February 2021, information on race was available for 21,005 (82%) patients and 449 (>99%) deaths. Information from these records was used to calculate incidence (cases per 100,000 population) and mortality (deaths per 100,000 population) and corresponding 95% confidence intervals (CIs) by population group. Population estimates were calculated using data from the U.S. Census Bureau.^†††^ Analyses were conducted using SAS (version 9.4; SAS Institute). To maintain patient privacy, numbers of cases or deaths among racial groups were not reported when the number of cases or deaths was less than 10; rates were not calculated when less than 20 cases or deaths were reported. This public health surveillance activity was reviewed by HDOH and CDC and was conducted consistent with applicable state and federal law and CDC policy.^§§§^^,^^¶¶¶^

During March 1, 2020–February 28, 2021, in Hawaii the COVID-19 incidence was 1,477 per 100,000 population and mortality rate was 32 per 100,000 population (Table). In aggregated analyses of incidence, Native Hawaiian and Pacific Islander persons experienced the highest incidences (2,501) across the five minimum race categories. In disaggregated analyses, Pacific Islander persons, who account for 5% of Hawaii’s population, represented 22% of cases. Pacific Islander persons had the highest COVID-19 incidence of 7,070; incidence among Native Hawaiian persons was 1,181. After further disaggregation, the highest incidence of cases among all Pacific Islander subcategories occurred among Marshallese persons (10,580), followed by Other Micronesian persons (8,991) and Samoan persons (4,525) (Figure). In disaggregated analyses of crude mortality, Pacific Islander persons experienced a crude mortality rate of 150 deaths per 100,000 population and accounted for 22% of deaths during this period. Mortality rate among Native Hawaiian persons was 15.

**TABLE:** Distribution of COVID-19 cases, incidence, deaths, and mortality rates, by race (alone or in combination with one or more other races)*^,^^†^ — Hawaii, March 1, 2020–February 28, 2021

Race**^§^**	Population**^¶^**(%)	No. of cases**^¶^**(%)	Cases per 100,000 population (95% CI)	No. of deaths**^¶^**(%)	Deaths per 100,000 population (95% CI)
**All races**	**1,422,094**	**21,005**	**1,477 (1,457–1,497)**	**449**	**32 (29–35)**
Native Hawaiian and Pacific Islander	369,956 (26)	9,253 (44)	2,501 (2,451–2,551)	145 (32)	39 (33–46)
Native Hawaiian**	304,167 (21)	3,591 (17)	1,181 (1,142–1,219)	45 (10)	15 (11–19)
Pacific Islander^††,§§^	65,789 (5)	4,651 (22)	7,070 (6,874–7,265)	99 (22)	150 (121–180)
Samoan	34,674 (2)	1,569 (7)	4,525 (4,306–4,744)	21 (5)	61 (35–87)
Tongan	7,855 (1)	190 (1)	2,419 (2,079–2,759)	<10^¶¶^ (<1)	—***
Other Polynesian	5,372 (<1)	54 (<1)	1,005 (739–1,272)	<10 (<1)	—
Guamanian or Chamorro	6,185 (<1)	59 (<1)	954 (712–1,196)	<10 (<1)	—
Marshallese	8,960 (1)	948 (5)	10,580 (9,944–11,217)	19 (4)	—
Other Micronesian	20,198 (1)	1,816 (9)	8,991 (8,597–9,386)	49 (11)	243 (175–310)
Fijian	816 (<1)	17 (<1)	–***	0 (—)	0 (—)
Other Melanesian	64 (<1)	<10 (<1)	–	0 (—)	0 (—)
Other Pacific Islander, not specified	3,725 (<1)	148 (1)	3,973 (3,346–4,600)	<10 (<1)	—
**Asian** ^†††^	802,551 (56)	8,807 (42)	1,097 (1,075–1,120)	272 (61)	34 (30–38)
Japanese	310,397 (22)	1,762 (8)	568 (541–594)	101 (22)	33 (26–39)
Filipino	367,291 (26)	4,579 (22)	1,247 (1,211–1,283)	108 (24)	29 (24–35)
Chinese	205,126 (14)	1,448 (7)	706 (670–742)	42 (9)	20 (14–27)
Korean	52,410 (4)	339 (2)	647 (578–716)	14 (3)	—
Vietnamese	14,998 (1)	180 (1)	1,200 (1,026–1,374)	<10 (<1)	—
**White**	611,108 (43)	5,790 (28)	947 (923–972)	52 (12)	9 (6–11)
**Black**	50,593 (4)	702 (3)	1,388 (1,286–1,490)	<10 (<1)	—
**American Indian or Alaska Native**	34,512 (2)	203 (1)	588 (508–669)	<10 (<1)	—
**Other race** ^§§§^	36,646 (3)	1,347 (6)	3,676 (3,483–3,868)	10 (2)	—

**Abbreviations:** CI = confidence interval; HDOH = Hawaii State Department of Health.

* Data analyzed included 21,005 (82%) of 25,480 cases and 449 (>99%) of 450 deaths, for whom information on race was available, reported to the HDOH during March 1, 2020–February 28, 2021. Incidence was calculated using the following equation: (cases/population) x 100,000 persons. Crude death rates were calculated using the following equation: (deaths/population) x 100,000 persons. 95% CIs were computed using normal approximation for standard errors for proportions. Population estimates were from the U.S. Census Bureau’s American Community Survey population estimates.

^†^ Data from race groups were examined without regard to ethnicity. Race information for cases was mostly self-reported; race information for deaths were reported by patients premortem, by an observer (e.g., physician), or by a proxy family member. Analyses were conducted with groups that were not mutually exclusive including persons of a race alone or in combination with one or more races. Using this approach, persons with more than one race indicated were included in the total of each race reported. Thus, all race categories (e.g., Asian) and subcategories (e.g., Filipino) consist of persons with any mention of those race categories or subcategories.

^§^ Alone or in combination with one or more races

^¶^ Category values do not sum to the total count or percentage because categories represent persons of a race alone or in combination with one or more other races. Subcategory values do not sum to category values for the same reason.

** This category includes persons identified as Native Hawaiian alone or in combination with another race.

^††^ This category includes persons identified as Pacific Islander alone or in combination with another race (e.g., this can include persons identified with both the Native Hawaiian race and a non-Native Hawaiian and Pacific Islander race). This category was calculated by identifying the proportion of population, cases, and deaths that remained from the total Native Hawaiian and Pacific Islander population after considering Native Hawaiian single race data.

**^§§^** Pacific Islander subcategories represent the populations among this group with the largest representation in Hawaii. Persons of more than one specific Pacific Islander race could be in more than one specific Pacific Islander race category. Pacific Islander persons with the Pacific Islander race category selected but who did not have a specific Pacific Islander race listed are included in the “Other Pacific Islander, not specified” category.

^¶¶^ <10 cases or deaths were reported; excludes zero. To maintain patient privacy, counts of cases or deaths among race groups were not reported when number of cases or deaths were <10.

*** Dashes indicate that rates were not calculated where <20 cases or deaths were reported.

^†††^ Asian subcategories represent the populations among this group with the largest representation in Hawaii.

^§§§^ Other race category includes persons with the “other” race category selected with no further specifications or with specified races that were not listed as a category (e.g., if a person had “Hispanic or Latino” indicated as their “race” or had written in a specific country).

**FIGURE:**
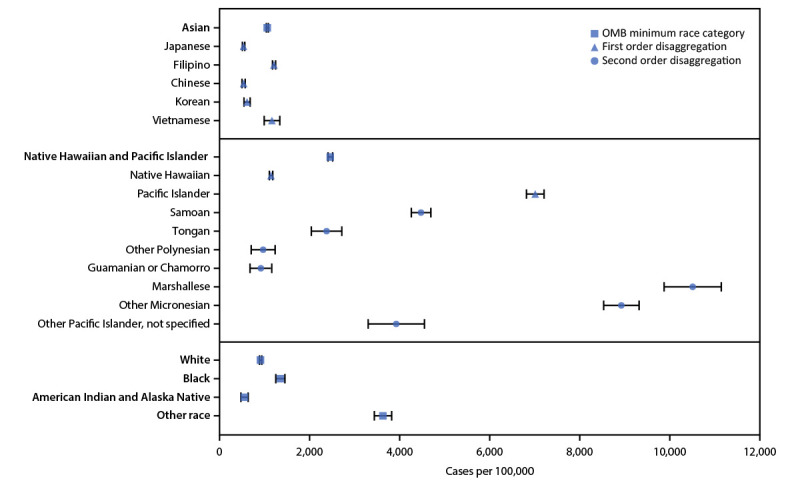
COVID-19 case rates,* by race (alone or in combination with one or more other races)^^†^^^^,^^^^§^^^^,^^^^¶^^ — Hawaii, March 1, 2020–February 28, 2021 **Abbreviations:** CI = confidence interval; OMB = Office of Management and Budget. * Case rates were based on COVID-19 cases reported to the Hawaii State Department of Health during March 1, 2020–February 28, 2021 and were calculated as (cases/population) x 100,000. Population estimates were from the U.S. Census Bureau’s American Community Survey population estimates. Data analyzed included 21,005 (82%) of 25,480 patients for whom information on race was available. Bars represent 95% CIs for the rates. † Data from racial groups were examined without regard to ethnicity. Analyses were conducted with groups that were not mutually exclusive including persons of a race alone or in combination with one or more races; persons of more than one race were included in the total for each race reported. Asian, American Indian or Alaska Native, Black or African American, Native Hawaiian and Other Pacific Islander, and White represent the five minimum race categories required by the OMB. Samoan, Tongan, Other Polynesian, Guamanian or Chamorro, Marshallese, Other Micronesian, and Other Pacific Islander, not specified represent subcategories within the Pacific Islander category. § Square markers indicate Other race or OMB’s five minimum race categories (American Indian or Alaska Native, Asian, Black or African American, Native Hawaiian or Other Pacific Islander, and White). ¶ Other race category includes persons with the “other” race category selected with no further specifications or with specified races that were not listed as a category (e.g., if a person had “Hispanic or Latino” indicated as their “race” or had written in a specific country).

Among Asian persons, there was also substantial variation in incidence among subgroups after disaggregation (range = 568 to 1,247 cases per 100,000 population). The highest incidence of cases among Asian persons were among Filipino persons (1,247) and Vietnamese persons (1,200); incidence among Japanese persons was 568. Among Asian subcategories, crude mortality rates ranged from 20 deaths per 100,000 population among Chinese persons to 33 among Japanese persons.

## Discussion

Disaggregation of COVID-19 data in Hawaii revealed substantial disparities in COVID-19 case and mortality rates during March 1, 2020–February 28, 2021, among Native Hawaiian, Pacific Islander, and Asian persons that were obscured in the aggregate data. Detailed information on disparities in COVID-19 cases and deaths among Marshallese persons has been reported (*2*,*8*); however, less information has been available regarding other Pacific Islander or Asian subgroups. These findings demonstrate the value of having access to disaggregated data at the state level to identify and reduce disparities and to provide relevant data to communities (*4*,*5*,*7*).

Collection of disaggregated surveillance data was recommended by local Native Hawaiian and Pacific Islander communities and grassroots groups early in the pandemic, resulting in the updating of the COVID-19 case report form by HDOH to collect these data. Patients with COVID-19 whose cases were reported before revision of the case report form were retrospectively contacted by HDOH staff members for detailed race information.**** During periods of higher incidence, HDOH continued to prioritize obtaining important demographic information, including race, even when conducting abbreviated case interviews. Efforts were designed to achieve a balance between highlighting the concerns of specific populations and inadvertently contributing to the stigmatization of groups who have been marginalized and who experience racism.

Race can serve as a marker for underlying systemic and structural inequities that drive health disparities. The COVID-19 pandemic underscores the need to prevent and reduce inequities in the social determinants of health, access to health care, and health conditions (*8*,*9*). There are simultaneous needs for advancing cultural responsiveness, language access, and sensitivity in public health strategies for preventing COVID-19 among Native Hawaiian, Pacific Islander, and Asian subgroups.^††††^ In Hawaii, disaggregation of COVID-19 surveillance data facilitated collaboration between HDOH and community partners equipped with culturally situated knowledge (*8*,*10*) to address disparities through tailored strategies.

HDOH created the Pacific Islander Priority Investigations and Outreach Team by engaging and training culturally responsive and linguistically diverse case investigators, contact tracers, and community health workers. The team includes staff members from the most affected Pacific Islander communities. This team provided translated prevention information, improved access to resources (e.g., isolation and quarantine facilities and comprehensive social services through community partners), and supported community outreach (e.g., providing interpretation assistance at testing sites). Prevention messaging incorporated cultural values and highlighted messages of protecting community; alternative strategies were encouraged for engaging in important cultural traditions and practices (e.g., cultivating collaborative partnerships to support virtual capacity for religious services). These efforts complemented efforts by advocate organizations and grassroots initiatives within Native Hawaiian, Pacific Islander, and Filipino communities.^§§§§^

The findings in this report are subject to at least six limitations. First, these data could underestimate COVID-19 case rates because of undetected cases and the exclusion of 18% of cases because data on race were missing. Second, case information was not available on characteristics such as occupation, income, and education, which can influence COVID-19 outcomes, and nativity and generational status, which might be associated with access to services and other social determinants of health. Third, the examination of disparities among specific combinations of categories (e.g., persons who are Samoan and White) was not possible because detailed U.S. Census data to calculate these rates were not available. Fourth, differences in the collection of race information between the case surveillance system and U.S. Census forms might have led to overestimation of rates among some race subgroups. For some races, race information was collected using explicit check-box options during case investigations, and in the U.S. Census, race information was collected through written-in free text that was later coded.^¶¶¶¶^ This could potentially lead to the reduction of rate denominators among specific race groups. Fifth, age-adjustment or stratification of rates could not be conducted because of lack of age-specific U.S. Census population information and limited sample sizes among specific Native Hawaiian, Pacific Islander, and Asian subgroups. Data on comorbidities, such as obesity, were also not available, limiting the ability to control for medical conditions which might vary across racial groups. Inability to incorporate age and comorbidities in analysis of mortality data could potentially lead to under- or overestimation of disparities in mortality rates.***** Finally, the use of race groups that were not mutually exclusive might limit the ability to make direct comparisons between groups because multiracial persons could be counted in more than one race group. Nonetheless, the use of race groups that were not mutually exclusive is advantageous when analyzing data among multiracial persons.

Substantial disparities in COVID-19 incidence and mortality rates during March 1, 2020–February 28, 2021, were identified through community-informed data disaggregation among Native Hawaiian, Pacific Islander, and Asian subgroups in Hawaii. The disparities identified among Marshallese, Other Micronesian, Samoan, Filipino, and Vietnamese persons, which were obscured in aggregated analysis, highlight the importance of partnering with these populations to develop culturally responsive outreach teams and tailored public health interventions and vaccination campaigns to more effectively address health disparities.

**Summary**

**What is already known about this topic?**

Aggregated race data can obscure health disparities among subgroups.

**What is added by this report?**

During March 2020–February 2021, community-informed data disaggregation in Hawaii indicated Pacific Islander persons, who account for 5% of the Hawaiian population, represented 22% of COVID-19 cases and 22% of COVID-19–related deaths. Among Asian populations, the highest COVID-19 incidences occurred among Filipino and Vietnamese persons.

**What are the implications for public health practice?**

Disaggregating race data can aid in identifying racial disparities among specific subpopulations and highlights the importance of partnering with communities to develop culturally responsive outreach teams and tailored public health interventions and vaccination campaigns to more effectively address health disparities.

## Footnotes

* These authors contributed equally to the report.

† Native Hawaiian persons are indigenous Hawaiians with ancestry to the original inhabitants of these islands. A majority of Pacific Islander persons in Hawaii are Samoan, Tongan, Chamorro or Guamanian, Chuukese, Palauan, and Marshallese persons. The latter three Pacific Islander groups migrated from the Federated States of Micronesia, Palau, and the Marshall Islands through provisions of their respective Compacts of Free Association. Immigration of Filipino persons to Hawaii from the Philippines began in the early 1900s when Filipino persons were recruited for agricultural labor.

§
https://www.cdc.gov/coronavirus/2019-ncov/community/health-equity/race-ethnicity.html#anchor_1595551025605

¶
https://healthpolicy.ucla.edu/publications/Documents/PDF/2021/COVID-19-Data-NHPI-Asians-factsheet-may2021.pdf

**
https://48ada3fb-53b7-4311-b1dc-3087b402628b.filesusr.com/ugd/11aeb5_4c461b06f90843a8ba2188dfe1c7e36a.pdf

†† COVID-19 cases included persons who received a laboratory-confirmed positive reverse transcription–polymerase chain reaction (RT-PCR) test result for SARS-CoV-2. COVID-19 deaths included decedents who had received a positive RT-PCR test result and had COVID-19 listed as a cause of death in the death certificate, discharge summary, or coroner’s notes.

§§
https://hawaiicovid19.com/wp-content/uploads/2021/03/COVID-19-Race-Ethnicity-Equity-Report.pdf

¶¶
https://www.govinfo.gov/content/pkg/FR-1997-10-30/pdf/97-28653.pdf

*** In 2019, 24.2% of Hawaii’s population was multiracial, identifying as two or more races using OMB minimum race categories (https://census.hawaii.gov/wp-content/uploads/2020/06/Hawaii-Population-Characteristics-2019.pdf;(https://www.census.gov/prod/cen2010/briefs/c2010br-12.pdf). Using OMB minimum race categories, 19% of cases and 8% of deaths had two or more races indicated; when allowing for specific Native Hawaiian, Pacific Island, and Asian races, 21% of cases and 10% of deaths had two or more races indicated.

††† Population estimates for “race alone and in combination with one or more other races” were from the U.S. Census Bureau’s American Community Survey population estimates. https://www.census.gov/data/developers/data-sets/acs-5year.html

§§§
https://health.hawaii.gov/docd/files/2017/01/HAR-Title-11_Chapter-156.pdf

¶¶¶ 45 C.F.R. part 46.102(l)(2), 21 C.F.R. part 56; 42 U.S.C. Sect. 241(d); 5 U.S.C. Sect. 552a; 44 U.S.C. Sect. 3501 et seq.

**** Case information was collected through three possible mechanisms including either a provider form (revised version https://health.hawaii.gov/docd/files/2020/01/COVID-19_Short-Form_Fillable_For_Physicians.pdf), case investigation form, or the HDOH case surveillance system (which uses the CDC Public Health Race Value set: https://phinvads.cdc.gov/vads/ViewValueSet.action?id=67D34BBC-617F-DD11-B38D-00188B398520). For the provider and case investigation forms, persons who provided Pacific Islander race or Other Asian race were given the opportunity to specify which specific Pacific Islander or Asian race with which they identified. Persons with race indicated as Pacific Islander or Filipino race were followed up with by the HDOH Pacific Islander Priority Investigations and Outreach Team.

††††
https://www.federalregister.gov/documents/2021/01/29/2021-02073/condemning-and-combating-racism-xenophobia-and-intolerance-against-asian-americans-and-pacific?utm_medium

§§§§ Advocate organizations and grassroots initiatives within Native Hawaiian, Pacific Islander, and Filipino communities included the Native Hawaiian and Pacific Islander Hawaiʻi COVID-19 Response Recovery and Resiliency Team. (https://www.nhpicovidhawaii.net/) and the FilCom CARES project (https://www.filcomcares.org), among others.

¶¶¶¶ Specific Pacific Islander groups coded in the U.S. Census based on free-text responses are “Tongan,” “Other Polynesian,” “Marshallese,” “Other Micronesian,” “Fijian,” and “Other Melanesian.”

***** Unpublished analysis of COVID-19 mortality data from Hawaii suggests that age adjustment of mortality rates results in more pronounced disparities for COVID-19 mortality among populations with younger age distributions (e.g., Native Hawaiian and Pacific Islander persons) compared with populations with older age distributions (e.g., Japanese or White persons).
